# Ebola, quarantine, and the need for a new ethical framework

**DOI:** 10.18502/jmehm.v13i9.4078

**Published:** 2020-08-26

**Authors:** Corey Benjamin Moore

**Affiliations:** *School of Public Health and Community Medicine, University of New South Wales, Sydney, Australia.*

**Keywords:** Quarantine, Ethics, Ebola virus.

## Abstract

Quarantine is a broad public health strategy used to control infectious diseases outbreaks. An arguably most aggressive public health intervention, quarantine limits the asymptomatic individuals’ liberty and can result in significant harm. Quarantine was used in an attempt to control several Ebola outbreaks during the Ebola epidemic in West Africa in 2014. The most concerning quarantine intervention occurred at West Point, a slum of 75,000 people in the capital Liberian capital, Monrovia. This work critically reviews present ethical frameworks in public health for the examination of outbreaks in West Africa. This work utilizes the nine public health ethical principles described by Kerridge, Lowe and Stewart to argue that the quarantine at West Point was not ethically justified; and, it concludes that a new ethical framework for quarantine is required to address future outbreaks in the West African context.

## Introduction

Quarantine is a broad public health strategy attempting to contain disease outbreaks by stopping communicable diseases’ transmission chain through limiting, isolating and monitoring contact of individuals who have been or who may be exposed to the disease  (1, 2) . Quarantine is one of several public health strategies to prevent the communicable diseases’ spread. It originates from general moral obligations to prevent harm to others; and, it is arguably the most aggressive public health measure as it limits the asymptomatic individuals’ liberty (3) . Quarantine raises conflicting ethical values as it creates and worsens unequal burdens and benefits among individuals and groups. Both ethical values and decisions reflect and are influenced by cultural differences and political pressures.

Ebola virus disease (EVD) is a communicable disease transmitted through direct contact with bodily fluids. It was first raised in 1976 and it has caused ten outbreaks since then  (4, 5) . No vaccine or pharmacological treatment exists for EVD (6) . In March 2014, WHO declared an EVD outbreak in West Africa, a public health emergency of international concern (7) . The main affected countries were Liberia, Sierra Leone and Guinea. These countries have a history of unstable government, political violence, civil war, social disadvantage, and weak public health infrastructure  (8-10) . These conditions contributed to a rapid spread of outbreak (11)  and made military-enforced quarantine inevitable (8) . The most concerning quarantine intervention was at West Point, a slum of approximately 75,000 people in the Liberian capital, Monrovia. Military troops were employed to restrict movement  (2, 8) . The quarantine, however, was not effective in the outbreak control (12)  and ended after ten days of civil unrest (13) . 

***Rapid changes imposed on quarantine during crisis***

 Although international law supports the right to freedom of movement (14) , all legal systems and international human rights permit quarantine (15) . The specifics and use of quarantine vary by country; however, public health officials are allowed to use measures to protect public health (16) . While few countries have ethical frameworks to guide difficult decision-making for epidemics (17) , the existing legislation and guidelines of democracies reflect their citizens’ ethical values (18) .

Public health interventions such as quarantine should be exercised according to the law and with appropriate evidence; however, during a crisis these laws can change quickly and be enforced arbitrarily, often based on community fear and political pressure  (13, 19) . For example, during the EVD epidemic, Canada denied visas to individuals from countries with EVD outbreaks, against international health regulations (20) . In USA, quarantining of healthcare workers who had worked in West Africa was inconsistent and politically motivated (21) . During the avian flu pandemic, Australia stockpiled $192m on an antiviral with questionable efficacy (22) , and the fear caused by the EVD epidemic contributed to the creation of a $300m centre to manage future epidemics (23) .

***Public health ethical frameworks to study quarantine***

A number of public health frameworks exist that address ethical issues of public health interventions. However, the broadness of the issues that public health need to address makes achieving a generalizable ethical framework difficult (24) . The application of such frameworks to quarantine is even more difficult given the quarantine’s potential in limiting autonomy, causing harm, targeting vulnerable people and exacerbating inequalities. 

 Some public health frameworks need to justify the effectiveness of an intervention. For example, the ethical framework described by Kass   states that in case of a lack of evidence, the intervention is questionable and should be terminated (25). However, at the beginning of an outbreak, often insufficient information is available to determine if a quarantine will be effective (1) . Such frameworks may prevent action due to a lack of evidence, which could lead to worse outcomes.

For the abovementioned reasons, ethical frameworks have been developed specifically for quarantine  (13, 26, 27) ; however, these as well as others can suffer from ideological and cultural biases. For example, Kass   gives increased weight to liberty (25), Baum   argues for less emphasis on autonomy (28), Calman and Downie   for increased utility (29), and Gostin et al.   states the importance of individual principles and is not sensitive to cultural traditions  (26,30-32) . Such liberal views may not be relevant for addressing issues in Africa (30) . Therefore, ethical principles that are flexible, less prescriptive and have less Western biases are more appropriate for addressing public health interventions in an African context. 

The public health ethics’ principles described by Kerridge et al.  have been derived from many ethical discussions on the appropriateness of public health interventions (31). They do not put weight or bias on any single principle, and thereby reduce Western values’ influence (31) . Hence, they are considered appropriate in analysing the quarantine ethics at West Point.

***Necessity for a feasible and effective quarantine***

Quarantine has been successfully used as a public health strategy in preventing the disease spread in past outbreaks (e.g., influenza (33) , measles  (34)  and SARS (35) ). However, at the time of an outbreak, evidence for the effectiveness of quarantine is often quite limited  (36) . Therefore, the level of evidence required to justify quarantine must be much lower than that of other public health interventions due to the potential harm of the disease to the public. Hence, precautionary principle, beliefs and ethical recommendations are utilized to justify the use of quarantine in the absence of quality evidence  (24, 26) . However, feasibility, an important aspect of effectiveness, can be overlooked, and may be known with some certainty at the beginning of an outbreak. If a quarantine is not feasible, it does not meet the principle of effectiveness, described by Kerridge et al. (31) . Hence, feasibility needs to be considered in evaluating the effectiveness of a quarantine.

Conditions triggering disease outbreaks such as socioeconomic inequality and weak health infrastructure have been used to justify the extreme public health intervention of quarantine  (5, 18) . However, these conditions also limit the surveillance capacity, contract tracing, public trust and health education, which are all critical to maintain stability during a quarantine, and to identify and isolate the infected individuals  (6, 37) . Quarantine, imposed under these conditions, would not only do require people to give up their liberty, but would also put them at increased risk of infection harm and further inequalities. Despite this, a consequentialist argument could justify such harm if containing a disease outbreak and preventing harm to the greater community were possible. However, under such inadequate conditions, an intervention is unlikely to prevent harm to the greater community because those quarantined are unlikely to support and comply with quarantine measures. Such circumstances was the case at West Point. Military-enforced quarantine did not benefit the greater community, thereby resulting in increased harm to those quarantined and creating civil unrest leading to breaches and the eventual abandoning of the intervention (13) . 

***Few public health interventions with weak public health infrastructure***

Quarantine may be justified if public health is threatened  and less restrictive strategies (e.g., education, monitoring, decreased social mixing and increased social distancing) cannot achieve an appropriate outcome (31) . In the case of EVD, symptoms develop in individuals before they become infective (38) ; hence, anyone with symptoms could potentially be identified and isolated before becoming a risk to others. That is, given appropriate resources and procedures, measures less restrictive than quarantine can achieve the same goal of preventing EVD spread. 

However, in Liberia, a lack of public health infrastructure meant that these less restrictive measures were not an option, thereby effectively limiting initial public health options to nothing or quarantine. WHO had recommended against quarantine (2) ; however, considerable local and international pressures demanded the politicians and public health officials to stop the epidemic in some way  (4, 39) . Similar pressures also resulted in quarantine in Western neoliberal countries as well (8) .

***Counter-productivity of excessive infringement***

Public health interventions should be implemented with the least infringement  (24) . In the case of quarantine, measures range from voluntary movement restriction to military-imposed restriction. This principle respects individuals’ and community’s liberties, rights, interests and needs (39) . This is crucial as those most affected by quarantine are at risk of misappropriation of restrictive measures, thus requiring special efforts to protect them (39, 40) .

The use of the military to implement quarantine in resource-poor countries with weak public health structures is often inevitable (8) . Strong coercive measures are rationalized because voluntary compliance is unlikely considering the lack of public consultation, acceptance, and reciprocity. In addition, public health infrastructure limits the ability to implement less restrictive measures, such as using web-cameras to monitor for symptoms as was done in the quarantine in Singapore during the SARS pandemic (41) . The military-enforced quarantine in Beijing during SARS saw 250,000 flee the city overnight (42)  and the quarantine in Taiwan was counter-productive due to the panic it created (18) . At West Point, the use of the military to enforce quarantine resulted in distrust, decreased compliance, worse health outcomes and increased stigma. Hence, the quarantine did not meet the least infringement principle.

***Necessity for community consultation***

Public health decisions should be justified through discussion with the community (31, 43) . These discussions and subsequent decisions should be made without political interference and with fair representation (24)  while concerning transparency, due process and fairness factors (42) . However, this is difficult due to the social and political relationships of power existing within communities (37) . It is also difficult during public health emergencies because such consultation consumes already-limited public health resources especially in low-income, developing countries. The authoritarian history and culture of some countries such as Liberia also make it challenging. Nonetheless, public health actions need to be understood by the affected people and consistent with local values. 

Interventions also need public trust as they require individuals to follow orders that are counter-productive to their personal benefit (18) . This trust should be built during good times, and then maintained and protected during interventions (31) . Mistrust of government and public health officials at the beginning of an epidemic are also worsened with the use of military (21) . At West Point, this distrust was counter-productive to the interventions’ intentions. The use of humanitarian organisations to enforce restrictive measures created a perceived conflict of interests, resulting in further distrust (8) . People were afraid of reporting symptoms and preferred to remain under their families’ and friends’ care, thereby spreading the disease (9) ; individuals thought public health measures were being made by foreigners in exchange for increased personal wealth of officials (10) ; a screening centre was destroyed because people believed that the government was bringing sick people into their community; soldiers were bribed to leave the quarantine area and citizens were shot (13) . After ten days; these actions and civil unrest resulted in the quarantine cancellation (13) . 

***Quarantine reflecting community core values and culture***

Public health interventions need to be aligned with community values to maintain social structures during and after quarantine  (13, 31, 44) . Ethical questions and their answers are subject to cultural differences (37) . Quarantine has been argued to be less tolerable in liberal states assigning a greater weight on liberty (13) . However, quarantine measures can contradict deep cultural beliefs, thus making it an intolerable measure in societies with more communitarian values. In addition, quarantine can question and stigmatize cultural practices. 

Cultural practices in West Africa involve the family and community of the dying being included in their care and intimate traditional customs in the preparation of the dead for the afterlife (6) . The Liberian government did not respect such practices; they denied contact of those identified as infected and enforced cremation of the dead. Consequently, people did not report if they had symptoms and preferred to stay at home under their families’ and friends’ care (9) . Such attitude created stigma of cultural practices and reduced social cohesion. Since the intervention was not aligned with the community values, individuals’ moral obligation to report and comply with the quarantine remains questionable. 

***Community’s moral obligation to protect affected individuals***

Those under quarantine are more likely to be the community’s vulnerable members and are at the greatest risk of harm from public health interventions. They are asked to relinquish their liberty and face hardship to benefit the greater public good (13) . The unaffected part of community then has a responsibility to prevent further exacerbation of hardship and allocate resources to compensate for loses. During the SARS epidemic, Taiwan provided fixed sum compensation (45), Canada provided income replacement (46) , and Singapore provided criminal immunity to increase public health orders’ compliance (41) .

Furthermore, to compensate for immediate and direct harm, protecting against stigmatization and its sequel is necessary (1) . Stigma, a major social determinant  (47) , resulted in job losses and rejection from community in West Africa  (48, 49) . In Nigeria, psychosocial teams held meetings in communities to reduce stigma (49) . In Canada, during the SARS quarantine, legislation prohibited discrimination against those quarantined (50) .

The quarantine in West Point targeted the vulnerable, and a lack of reciprocity (21) (i.e., those quarantined had no moral obligation to comply with the quarantine) resulted in worse health outcomes, starvation, financial losses and stigmatization’s long-term consequences. 

**Summary**

This paper has argued that the West Point quarantine was not ethical because it did not meet the nine ethical principles outlined by Kerridge et al. (31)  due to the followings:

1. Considering the outset, the quarantine was not effective since the outbreak had spread outside of the quarantined area (e.g., a lack of public health infrastructure made it infeasible), and since those quarantined were unlikely to comply with quarantine measures.
2. The quarantine failed the proportionality test as it caused harm to those quarantined and did not protect the majority of population.
3. The lack of infrastructure meant unavailability of less invasive public health interventions. Hence, the quarantine was not justified based on the principle of necessity because doing nothing would have been less invasive and less harmful.
4. The restrictive infringement involving use of military force was likely to have been counter-productive.
5. Insufficient evidence of any or adequate community consultation.
6. The taken actions exacerbated high mistrust levels.
7. The intervention targeted a vulnerable population and did not respect community and cultural values.
8. Inadequate support was provided for the individuals under quarantine, people exposed to worse health conditions, suffering financial hardship, facing increased stigma and exacerbated inequality.

The intervention did not allow for appeal, due practice or evaluation, and only ceased under political pressure after civil unrest (13) . 

## Discussion

***Necessity for a new framework***

This paper used the principles of public health ethics outlined by Kerridge, Lowe and Stewart (31)  to examine the quarantine at West Point because no existing ethical framework addresses quarantine as a special public health intervention and in the unique context of West Africa (34). Previous work have used existing public health ethical frameworks to examine the quarantine at West Point and have concluded that the quarantine was unethical  (8, 39, 51) ; in more marginal cases, biases or omissions in existing ethical frameworks may be important. Inevitability of future outbreaks in West Africa, the unavoidable use of quarantine to attempt to contain outbreaks, and similarities among the West African countries’ values (52)  make attaining such a framework specific to quarantine and West Africa both necessary and feasible.

Such a framework would address the influence of groups outside of the community, including other countries. Many of the issues requiring public health interventions in West Africa exhibit global injustice symptoms, and hence sharing the burden is an ethical responsibility of other countries. A new framework would consider the principle of proportionality on a more global level and consider sharing the burden of epidemics with other nations. While other nations did contribute to the control of the EVD epidemic in West Africa, this contribution was largely due to self-interest and did not result in fair reciprocity to the people directly affected by EVD. The original ethical considerations made by WHO were on questions of experimental pharmaceuticals since they were, arguably, more relevant to the donor nations’ interests (8) . Finally, frameworks have been created around more communitarian values and social solidarity as a result of recent epidemics (e.g.,  (13, 27) ); nevertheless, they do not adequately address differences across and between communities, weak or non-existent public health infrastructure’ implications, or cultural practices that are important for social cohesion and support, but contrary to quarantine measures. All of the abovementioned factor are major issues in West Africa.

## Conclusion

The existing ethical frameworks for considering and designing a quarantine intervention in West Africa do not adequately address the West Africa’s social and cultural values. Furthermore, they fail to consider the responsibilities of other communities that have contributed to the need for public health interventions. Since future outbreaks are inevitable, developing new ethical public health frameworks is required. The endorsement of such frameworks may encourage research and investment to prevent the conditions contributing to such outbreaks.

## Notes:

**Citation to this article:**

Moore CB. Ebola, quarantine, and the need for a new ethical framework. J Med Ethics Hist Med. 2020; 13: 9.
